# An organ systems-based review of outcomes associated with sleep apnea in hospitalized patients

**DOI:** 10.1097/MD.0000000000026857

**Published:** 2021-08-27

**Authors:** Maaz Sheikh, Stephen Kuperberg

**Affiliations:** a Stony Brook University Hospital, Department of Internal Medicine, Stony Brook, NY; b Stony Brook University Hospital, Department of Pulmonology and Critical Care, Department of Internal Medicine, Stony Brook, NY.

**Keywords:** inpatient outcomes, obesity hypoventilation, positive airway pressure, severe acute respiratory syndrome coronavirus 2, sleep apnea

## Abstract

The current global health crisis due to severe acute respiratory syndrome coronavirus 2 (SARS-CoV-2) has prompted the medical community to investigate the effects of underlying medical conditions, including sleep-disordered breathing, on inpatient care. Obstructive sleep apnea (OSA) is a common form of sleep-disordered breathing that may complicate numerous acquired conditions, particularly in inpatient and critical care settings. Viral pneumonia is a major contributor to intensive care unit (ICU) admissions and often presents more severely in patients with underlying pulmonary disease, especially those with obesity and OSA. This review summarizes the most recent data regarding complications of both OSA and obesity and highlights their impact on clinical outcomes in hospitalized patients. Additionally, it will highlight pertinent evidence for the complications of OSA in an organ-systems approach. Finally, this review will also discuss impatient treatment approaches for OSA, particularly in relation to the SARS-CoV-2 pandemic.

## 1 Introduction: Obstructive sleep apnea is a risk factor for hospitalization

Obstructive sleep apnea (OSA) is a widely prevalent condition characterized by repeated collapse of the airway during sleep, leading to significant morbidity and mortality.^[1,2]^ Physiologically, it is the result of pharyngeal collapse leading to partial or complete cessation of airflow during sleep.^[3]^ When occurring together with oxygen desaturation, such episodes can be qualified as apnea (total cessation of airflow for more than 10 seconds) or hypopnea (reduced airflow for more than 10 seconds).^[4]^ Measurement of severity is obtained via polysomnography and quantified using the apnea-hypopnea index, which represents the sum of apneas and hypopneas over a quantified period of sleep. An apnea-hypopnea index ≥5 correlates with mild disease, and ≥30 correlates with severe disease. Established disease associations include hypertension, metabolic syndrome, arrhythmias, heart failure, cerebrovascular disease, and mortality.^[1,3,5,6]^ The systemic effects of OSA on hospitalized patients are summarized in Table 1. Although multiple pathophysiological mechanisms underlie poor outcomes in OSA, the core feature is repetitive arousal during fragmented sleep that leads to intermittent hypoxia and hyperactivation of the sympathetic nervous system. Hypoxic events promote a cycle of ischemia and reperfusion, resulting in the transcription of pro-inflammatory cytokines and transcription factors, free radical injury, endothelial dysfunction, and cardiac ischemia and remodeling.^[7,8]^

**Table 1:** Obstructive sleep apnea can have downstream impacts on numerous organ systems in inpatients. These are summarized in this table.

Organ system	Morbidities associated with OSA in hospitalized patients
Pulmonary	Ventilatory insufficiency (causing daytime hypercapnia and chronic hypoxia), difficult intubations, pulmonary hypertension, respiratory failure
Cardiovascular	Systemic hypertension, congestive heart failure, stroke, tachyarrhythmias (primarily atrial fibrillation), sick sinus syndrome
Renal	Acute kidney injury
Neuropsychological	Stroke, memory deficits, delirium, poor concentration, mood changes

Downstream of these events is a broad spectrum of organ dysfunction seen in hospitalized medical and surgical patients. The role of OSA in worsening perioperative outcomes has been established in the surgical literature.^[9]^

The role of underlying OSA in hospitalized patients is especially important because of the severe acute respiratory syndrome coronavirus 2 (SARS-CoV-2) pandemic. While there is minimal literature to date that thoroughly defines a causal relationship between OSA and worsening SARS-CoV-2 outcomes, several studies have indicated that this suspected relationship is worth studying. The CORONADO study found a significant association between patients who were receiving treatment for OSA and their primary outcome studied (death within 7 days of admission).^[10]^ In a systematic review, Miller and Cappuccio discuss multiple studies that conclude multiple risk factors for OSA (including obesity, hypertension, and diabetes) result in worsening outcomes for SARS-CoV-2.^[11]^

## 2 Methods

All studies referenced in this review were obtained from the electronic databases PubMed (National Library of Medicine), Web of Science, and EMBASE. The articles that were considered for this review included observational studies, retrospective and prospective clinical trials, case reports, and meta-analyzes pertaining to the downstream systemic effects caused by OSA and obesity specifically in hospitalized patients. In particular, the outcomes of morbidity, mortality, ICU admission, and hospital length of stay were analyzed. Additionally, the same article types were included if they pertained to OSA and its effects on these outcomes in patients hospitalized with viral or bacterial pneumonia. Clinical studies pertaining to the effects of obesity on patients hospitalized with SARS-CoV-2 were also included. At the time of this study, the effects of OSA have not been well studied in its effects on patients with SARS-CoV-2. Keywords used for searching the database included “sleep apnea cardiovascular disease,” “sleep apnea neurological effects,” “sleep apnea inpatient systemic effects,” “sleep apnea pneumonia,” “sleep apnea obesity overlap syndrome,” “obesity COVID-19,” “obesity SARS-CoV-2,” and “sleep apnea sepsis.” Studies that minimized the risk of bias were included, and those that did not account for bias were excluded. Conclusions regarding the role of OSA in hospitalized patients were made based on the results of each of the studies that met the inclusion criteria.

### 2.1 Role of obesity

Obesity, defined as a body mass index (BMI) greater than 30 kg/m^2^, is the most commonly attributed underlying risk factor attributed to patients with OSA, accounting for 40% to 60% of cases.^[7]^ The epidemiologic significance of OSA is amplified by the burden of obesity in the population as a whole. The World Health Organization estimates that the global prevalence of obesity is 13%.^[12]^ An essential link exists between OSA and obesity, with an estimated 41% to 78% of those with OSA being clinically obese.^[13]^

A mismatch in the severity of illness and mortality in hospitalized patients is attributed to the “obesity paradox,” wherein patients classified as overweight or moderately obese have lower in-hospital and ICU mortality rates^[12–14]^; however, the biological mechanism of this paradox is not completely understood. An exception is in the morbidly obese with BMI > 40 kg/m^2^, in whom the length of stay, but not the mortality rate, is increased.^[14]^ The pathophysiology of this phenomenon is not yet clear,^[15]^ although Garrouste-Orgeas et al postulate that there may be an associated nutritional reserve that provides a source of metabolic needs in critically ill patients with a BMI greater than 30 kg/m^2^, especially when compared to patients with a BMI less than 18.5 kg/m^2^.^[16]^ However, the lack of clarity in this area calls for future investigation.

In contrast to this paradox, in which obesity was studied independently, overlap syndromes are more clearly associated with poor hospital outcomes. Obesity hypoventilation syndrome (OHS) is defined by a combination of BMI > 30 kg/m^2^, sleep-disordered breathing (SDB), and daytime hypercapnia (PaCO_2_ ≥45 mm Hg),^[17]^ and 70% of patients with OHS also have severe OSA. Overlap of OHS with OSA potentiates morbidity and is associated with higher rates of respiratory failure in hospitalized patients.^[18]^ Nowbar et al prospectively studied 150 hospitalized patients, finding that those with obesity-associated hypoventilation were more likely to require longer lengths of stay, ICU admission (40%), and long-term care at discharge and to have increased mortality compared to those with simple obesity.^[19]^ Ventilatory insufficiency in these patients stems from reduced lung compliance in conjunction with airway narrowing secondary to OSA, leading to acute worsening of chronic hypercapnia and hypoxia.^[20,21]^ Zerah et al found a link between airway conductance and FRC in obese patients.^[22]^

Positive airway pressure (PAP) is considered the mainstay of treatment for patients with OSA.^[3,23]^ Although the effects of PAP on inpatient mortality remain unclear, controversy exists regarding its benefits. For example, a meta-analysis of 10 trials by Yu et al found that the use of continuous positive airway pressure (CPAP) was not associated with improved cardiovascular outcomes (acute coronary syndrome events, stroke, cause-specific vascular events, and death).^[24]^ while another meta-analysis by Patil et al reviewed 184 studies and found that treatment with PAP significantly reduced disease severity, daytime sleepiness, and blood pressure. The authors noted that nonrandomized data showed that PAP was associated with reduced cardiovascular outcomes, but randomized data were not.^[25]^

CPAP utilization for OSA in hospitalized patients is low, resulting in no extractable data regarding inpatients.^[26]^ Spurr et al found that CPAP was utilized in only 5.8% of patients with OSA in the inpatient setting.^[27]^ Sorscher et al hypothesize that there may be multiple factors contributing to this, such as nursing unfamiliarity with using the machines, low availability of CPAP in the hospital, and inpatient providers considering OSA a nonurgent medical problem.^[26]^

Despite these controversies, CPAP is established as the primary treatment for OSA based on the American Academy of Sleep Medicine guidelines and is effective in reversing known cardiovascular and neurologic sequelae, such as hypertension. The ATS 2019 guidelines recommend CPAP as the primary treatment for OHS patients with severe OSA.^[17]^

### 2.2 Respiratory implications of obstructive sleep apnea in the inpatient setting

Intubation success is a special concern for critically ill patients with OSA. Due to the prevalence of obesity in this population, there are corresponding anatomical and pathophysiological barriers to intubation and oxygenation. A retrospective study by Siyam and Benhamou found that the incidence of difficult intubation in patients with OSA was 21.9% compared to only 2.6% in patients without OSA.^[28]^ Craniofacial and airway dysmorphology, such as short neck and narrowed airway diameter, constrains intubation success and may lead to intubation failure.^[29]^ This is exemplified in a study performed by Walsh et al, which found that patients with OSA had smaller velopharyngeal cross-sectional areas than patients without OSA.^[30]^ Obese patients are also more likely to possess decreased oxygen stores and impaired gas exchange due to atelectasis of the lung dependent zones.^[31]^ The mechanism behind this in paralyzed, sedated patients was elucidated in a study by Pelosi et al that compared the lung mechanics in ten morbidly obese postoperative patients matched with those of 10 nonobese postoperative patients. In this comparison, obese patients exhibited reduced lung compliance, reduced chest wall compliance, and reduced functional residual capacity.^[32]^

### 2.3 Pulmonary infections in obstructive sleep apnea and obesity

The EPIC I, EPIC II, and SOAP studies established that infections, particularly nosocomial infections, are associated with increased in-hospital mortality and longer lengths of stay.^[33–35]^ Pneumonia is identifiable in 20% of patients requiring hospitalization,^[36,37]^ anywhere between 10% and 19% require an ICU level of care,^[36–38]^ and it has an inpatient mortality rate ranging from as low as 22% to greater than 50%.^[37,39,40]^ This is especially applicable to patients with OSA, who are predisposed to respiratory infections due to body habitus, impaired cough reflex, and gastroesophageal reflux disease; as a group, they are also at increased risk for community acquired pneumonia, with severity of infection corresponding to severity of OSA.^[41]^ Vincent et al studied the effects of OSA on community acquired pneumonia in a retrospective cohort study, finding that underlying OSA is a risk factor for pneumonia.^[42]^ Su et al reported that the severity of OSA can indeed impact the severity of the infection.^[43]^ OSA has also been associated with higher initial rates of mechanical ventilation in patients hospitalized with pneumonia.^[44]^ A retrospective study by Lindenauer et al demonstrated that, in patients admitted to the hospital for pneumonia, those with OSA had an 18.1% likelihood of invasive ventilation and 28.8% of noninvasive ventilation. They also found that patients with OSA had higher risks for transfer to the ICU and an overall increased length of hospital stay.^[44]^

Viral pneumonia, especially in light of the ongoing SARS-CoV-2 pandemic, has long been a public health concern associated with high morbidity and mortality, particularly in obese patients.^[45]^ Numerous studies have confirmed the role of obesity as a strong independent risk factor for worse outcomes in patients with SARS-CoV-2,^[45–47]^ reflecting other pandemic viruses, including Middle Eastern Respiratory Syndrome (MERS) and H1N1 influenza.^[48,49]^

Seasonal influenza affects approximately 9 to 45 million people in the United States every year and has an estimated mortality of 12,000 to 61,000 annually.^[50,51]^ A meta-analysis performed by Coleman et al revealed that underlying conditions, including cardiovascular complications from obesity, rendered patients with influenza at a higher risk for ICU admission and at a higher risk for mortality. Among chronic respiratory diseases, asthma was associated with a similar increase in risk.^[52]^ Maier et al found an association between obesity and a higher incidence of viral shedding in patients diagnosed with influenza A.^[53]^ With respect to OSA specifically, a study by Beumer et al found that 11% of patients admitted to the ICU for influenza had underlying OSA compared to only 3% in patients not admitted to the ICU. Based on these findings it was concluded that OSA is an independent risk factor for ICU admission in influenza patients.^[54]^ The H1N1 global pandemic resulted in approximately 60.8 million cases, 274,304 hospitalizations, and 12,496 deaths in United States between April 12, 2009 and April 10, 2010.^[55]^ A cohort study performed by Webb et al analyzed ICU admissions in Australia and in New Zealand from H1N1 and found an ICU incidence of 28.7 per million habitants, the most prominent age group being 25 to 49. In addition, they found that 28.6% of ICU admissions had a BMI greater than 35 and 32.7% had a chronic pulmonary disease. Both of these values were far higher than the estimated disease prevalence in the general population.^[56]^

The World Health Organization states that PAP, a mainstay of treatment for OSA, is a high-risk aerosol-generating procedure that puts healthcare workers at risk.^[57]^ Society guidelines recommend withholding noninvasive positive pressure ventilation (NIPPV) in these patients, unless high-flow oxygen is unavailable.^[58]^ This poses a clinical dilemma, since the management of OSA with PAP is the standard of care. One strategy used is the use of viral heat and moisture exchange filters to reduce the aerosolized virus burden.^[59]^ The use of PAP as a means of treatment has also been described as potentially hazardous to healthcare workers in both SARS-CoV and MERS.^[60–62]^ In addition, patients diagnosed with MERS did not show improved outcomes when treated with NIPPV for respiratory failure.^[62]^ Similarly, and Yam et al found that there was no mortality benefit attributed to NIPPV with SARS-CoV.^[63]^ Whether to withhold NIV in SARS-CoV-2 continues to be debated, especially with regard to patients with obesity and OHS and those with overlapping OSA. In this setting, the clinical benefit of PAP must be balanced with aerosolization of the virus, posing a major risk to healthcare providers.

### 2.4 Extrapulmonary complications of obstructive sleep apnea in intensive care unit patients

**Figure 1:**
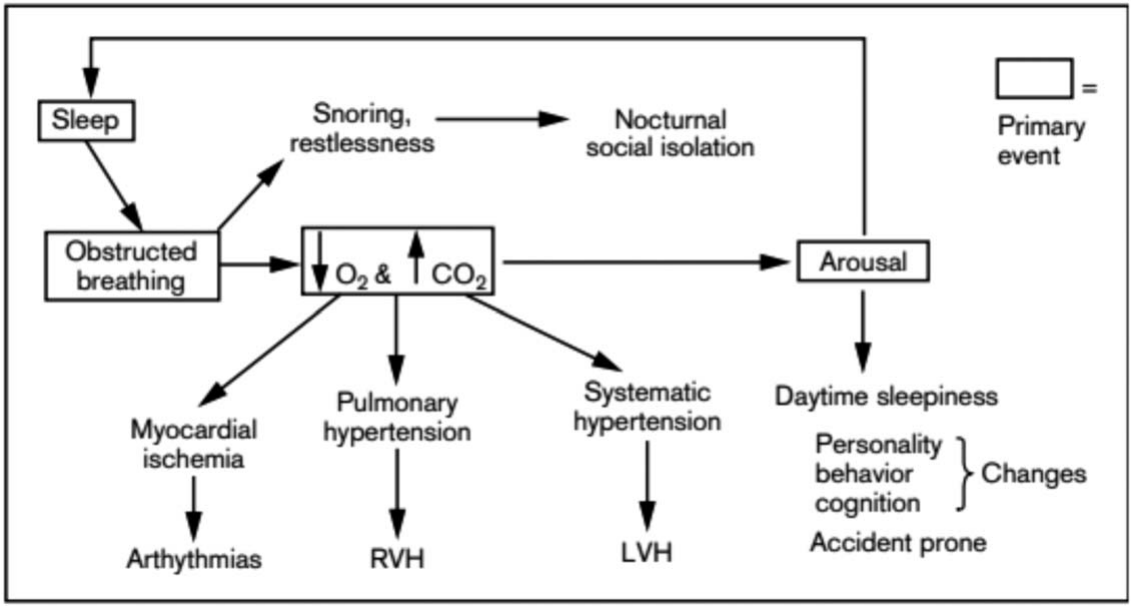
Systemic pathophysiology and downstream effects of obstructive sleep apnea. RVH = right ventricular hypertrophy, LHV = left ventricular hypertrophy. DOI:10.1097/01.aco.0000114685.04870.a4.

OSA, especially in severe cases, can affect multiple organ systems, as exemplified in Figure 1.^[29]^, and cardiovascular comorbidities in particular are well documented in association with OSA. OSA predisposes patients to congestive heart failure, likely as a result of hypoxemia, tachycardia, elevated left ventricular transmural pressure, and vascular injury.^[64–66]^ One mechanism postulated by Cofta et al proposed that OSA induces oxidative stress, leading to cardiovascular complications. This was done by measuring lipid peroxide levels in different degrees of OSA. The results showed a higher total antioxidant status and thiobarbituric acid-reacting substances in the more severe cases.^[67]^ It is also very likely that certain predisposition risk factors associated with obesity (i.e., hypertension, diabetes mellitus, etc.) contribute to these changes. Significant data also exist demonstrating that untreated OSA can result in both tachy and bradyarrhythmias. For example, atrial fibrillation (AF) has been associated with OSA in several studies, such as Gami et al, in which 151 patients with AF were compared with 312 patients without a diagnosis of AF. In all, 49% of the AF group was found to have OSA compared to only 32% in the non-AF group.^[68]^ Sick sinus syndrome and bradyarrhythmias appeared to have some association with OSA based on a prospective study by Almor et al that showed that 31.6% of patients requiring pacemaker placement for sick sinus syndrome had a diagnosis of OSA compared with the general population (3% prevalence of OSA).^[69]^ However, many of the studies correlating OSA with arrhythmias have small sample sizes, limiting extrapolation of the data.^[70]^

Organ injuries that are extrinsic to the cardiorespiratory system play important roles in critically ill patients with OSA. Central nervous system complications, particularly stroke, have been associated with untreated OSA. In fact, the prevalence of OSA in stroke patients is estimated to be 60% to 96%.^[23]^ A prospective study of 293 patients performed by Ahn et al found that SDB may be a risk factor for acute stroke. In this cohort of patients, 63.1% had a diagnosis of SDB.^[71]^ To further substantiate this correlation, cross-sectional and longitudinal analyses were performed on 1475 and 1189 people, respectively, by Arzt et al, revealing patients with moderate to severe OSA had a significantly higher risk for stroke than patients without SDB (odds ratio of 4.33).^[72]^

Neurocognitive complications, such as excessive daytime sleepiness, personality/psychosocial maladjustments, and mental impairment, are sequelae of intermittent hypoxia caused by the disordered breathing patterns associated with OSA, resulting in oxidative stress.^[73]^ Studies have found that patients with OSA demonstrate decreased gray matter volume in the orbital frontal cortex, frontal gyrus, hippocampus, and left cerebellum, leading to impairments in memory, cognition, and motor coordination.^[74,75]^ Furthermore, Canessa et al found that, after treatment with CPAP, patients showed improvement in cognition along with increased gray matter in the hippocampus and frontal structures.^[75]^

In a retrospective cohort study, renal complications of OSA in the ICU were investigated by Dou et al, assessing the ways in which OSA with systemic HTN impacts renal function over time, as it predisposes to risk for acute kidney injury during ICU admission. Using propensity-matched data, they found that 57% of ICU patients with OSA developed acute kidney injury compared with 46% in the non-OSA group, although they did not find any significant differences between the 2 groups in terms of length of stay and hospital mortality.^[76]^

## 3 Discussion

Obesity is considered a major risk factor for ICU admission and in-hospital mortality. Data suggest that, due to the strong association between OSA, obesity, and OHS and their synergistic effect on worsening outcomes, close attention should be paid to these often overlapping diagnoses in hospitalized patients. Anatomical and morphological changes render intubation more challenging and predisposed to decompensation. Strong evidence exists in the association of OSA with poor clinical outcomes in hospitalized patients, such as increased length of stay and cardiorespiratory, neurological, and renal complications, although a mortality risk has not been established.

Based on available data, it can also be concluded that patients hospitalized for pneumonia are at a higher risk for worse clinical outcomes when they have underlying OSA. Influenza is the only respiratory virus that has been studied thoroughly in relation to OSA, revealing a prolonged hospital length of stay but overall reduced mortality. In addition, growing evidence has confirmed that obesity poses a higher risk of worse outcomes in patients diagnosed with SARS-CoV-2.

OSA, especially in combination with OHS, affects multiple organ systems. Available evidence suggests that outcomes in hospitalized patients, particularly those with pneumonia and underlying cardiac disease, are affected. The use of CPAP may have an impact on morbidity; however, evidence for a mortality benefit is still lacking. This is of special concern in the current SARS-CoV-2 pandemic, where noninvasive ventilation, such as CPAP, poses a risk of aerosolization, and alternative management strategies must be sought.

## Acknowledgments

We would like to acknowledge the professional manuscript services of the American Journal Experts (AJE).

## Author contributions

**Investigation:** Maaz Sheikh.

**Methodology:** Maaz Sheikh.

**Project administration:** Stephen Kuperberg.

**Resources:** Maaz Sheikh, Stephen Kuperberg.

**Writing – original draft:** Maaz Sheikh.

**Writing – review & editing:** Maaz Sheikh, Stephen Kuperberg.
